# A Pyrazolo[3,4-*d*]pyrimidine compound inhibits Fyn phosphorylation and induces apoptosis in natural killer cell leukemia

**DOI:** 10.18632/oncotarget.11496

**Published:** 2016-08-22

**Authors:** Ilaria Laurenzana, Antonella Caivano, Stefania Trino, Luciana De Luca, Francesco La Rocca, Vittorio Simeon, Cristina Tintori, Francesca D'Alessio, Antonella Teramo, Renato Zambello, Antonio Traficante, Maddalena Maietti, Gianpietro Semenzato, Silvia Schenone, Maurizio Botta, Pellegrino Musto, Luigi Del Vecchio

**Affiliations:** ^1^ Laboratory of Preclinical and Translational Research, IRCCS Referral Cancer Center of Basilicata (CROB), Rionero in Vulture (PZ), Italy; ^2^ Department of Biotechnology, Chemistry and Pharmacy, University of Siena, Siena, Italy; ^3^ Biotecnologie Avanzate s.c.a.r.l., CEINGE, Napoli, Italy; ^4^ Department of Medicine Hematology and Clinical Immunology, Padua University School of Medicine, Padova, Italy; ^5^ Unit of Clinical Pathology, IRCCS CROB, Rionero in Vulture (PZ), Italy; ^6^ Department of Pharmacy, University of Genoa, Genova, Italy; ^7^ Scientific Direction, IRCCS CROB, Rionero in Vulture (PZ), Italy; ^8^ Department of Molecular Medicine and Medical Biotechnologies, University of Naples, Napoli, Italy

**Keywords:** natural killer large granular lymphocyte leukemia, Fyn tyrosine kinase, kinase inhibitor, NK cells

## Abstract

Natural killer (NK) cell neoplasms are characterized by clonal proliferation of cytotoxic NK cells. Since there is no standard treatment to date, new therapeutic options are needed, especially for NK aggressive tumors. Fyn tyrosine kinase has a key role in different biological processes, such as cell growth and differentiation, being also involved in the pathogenesis of hematologic malignancies. Our previous studies led us to identify 4c pyrazolo[3,4-*d*]pyrimidine compound capable of inhibiting Fyn activation and inducing apoptosis in different cancer cell lines. Here we investigated the presence of Fyn and the effect of its inhibitor in NK malignant cells. Firstly, we showed Fyn over-expression in NK leukemic cells compared to peripheral blood mononuclear cells from healthy donors. Subsequently, we demonstrated that 4c treatment reduced cell viability, induced caspase 3-mediate apoptosis and cell cycle arrest in NK cells. Moreover, by inhibiting Fyn phosphorylation, 4c compound reduced Akt and P70 S6 kinase activation and changed the expression of genes involved in cell death and survival in NK cells. Our study demonstrated that Fyn is involved in the pathogenesis of NK leukemia and that it could represent a potential target for this neoplasm. Moreover, we proved that Fyn inhibitor pyrazolo[3,4-*d*]pyrimidine compound, could be a started point to develop new therapeutic agents.

## INTRODUCTION

Natural Killer (NK) cell neoplasms are a rare and heterogeneous group of disorders characterized by excessive proliferation of cytotoxic CD3^−^ CD16/56^+^ NK cells. This group comprises two subtypes: aggressive NK leukemia (ANKL) and chronic lymphoproliferative disorder (CLPD) [1, 2]. ANKL is an Epstein Barr virus (EBV)-associated tumor most prevalent among Asian young adults (median age of 42 years). It has a fulminant clinical course, frequently resulting in death within two months. In contrast, CLPD has no demonstrable association with EBV and tends to occur in older adults (median age of 60 years). The clinical course is typically indolent, similarly to T-cell large granular lymphocytes leukemia (T-LGLL) [3–5].

Since no standard therapies for aggressive NK cell neoplasms have been established so far and the overall outcomes are dismal, new therapeutic options are needed.

Fyn, a tyrosine-specific phospho-transferase, is a member of Src family kinases which includes c-Src, Yes, Lck, Lyn, Hck, Fgr and Blk [6–8]. It phosphorylates a variety of target proteins involved in different signaling pathways [6]. Moreover, it regulates several biological functions, including growth factor and cytokine receptor signaling, cell-cell adhesion, integrin-mediated signaling, ion channel function, platelet activation, T and B-cell receptor signaling, axon guidance, mitosis, differentiation of NK cells [9–11].

In the last decade, the implication of Fyn in cancer biology and in hematologic malignancies has become more apparent. In chronic myeloid leukemia (CML) Fyn is up-regulated and its activation seems to be important in imatinib resistance [12, 13]. It is notably involved in the pathogenesis of peripheral T cell lymphomas [14] and in acute myeloid leukemia its higher expression, combined to FLT3-Internal Tandem Duplication (ITD), is correlated with poor prognosis [15].

The tissue-specific pattern of Fyn mRNA indicates that it is more expressed in normal NK and T cells respect to other human tissues [16].

Tintori *et al.,* by a structure-based drug design protocol and following hit-to-lead optimization, found 4c pyrazolo[3,4-*d*]pyrimidine compound driving inhibition of Fyn phosphorylation with a nanomolar range in an enzymatic cell-free assay. Moreover, the compound showed anti-proliferative activities against different cancer cell lines [17].

Therefore, in the present study we investigated the expression of Fyn in NK leukemic cells and the effect of 4c pyrazolo[3,4-*d*]pyrimidine compound in NK cell lines and in primary cells from chronic leukemic neoplasms.

## RESULTS

### Fyn is highly expressed in NK leukemic cells

**Figure 1:**
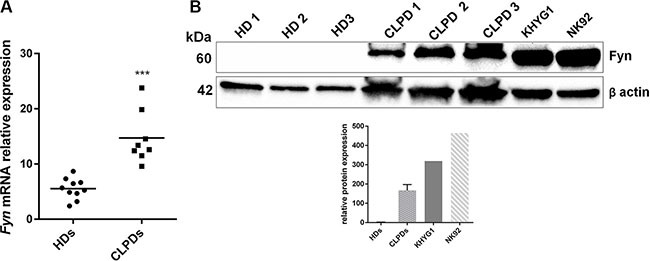
Fyn is over-expressed in NK malignant cells (**A**) qRT-PCR of *Fyn* mRNA in PBMCs from 10 HDs and from 8 patients with CLPD (UPN: 1–8). Statistical significance was determined using an unpaired *t* test and *P*-value is indicated by asterisk: ***p < 0.001. (**B**) WB analysis of Fyn in 3 HDs, 3 CLPD patients (UPN: 6–7) and two NK cell lines, KHYG1 and NK92. Quantification of Fyn protein levels was normalized with β-actin bands.

In order to quantify the presence of Fyn in NK leukemic cells, we firstly assessed its mRNA expression levels in peripheral blood mononuclear cells (PBMCs) from 10 healthy donors (HDs) and 8 CLPD patients by quantitative Real Time-Polymerase Chain Reaction (qRT-PCR). *Fyn* level was significantly up-regulated in PBMCs from CLPD patients compared to HDs (*p* < 0.001; Figure 1A). We also analyzed Fyn protein level by western blotting (WB) in PBMCs of 3 HDs, 3 CLPD patients and in two NK cell lines, KHYG1 and NK92. We observed a high level of Fyn protein in PBMCs from chronic patients and in NK leukemic cell lines respect to PBMCs from HDs (Figure 1B).

### 4c pyrazolo[3,4-*d*]pyrimidine compound reduced cell viability, induced apoptosis and cell cycle arrest in NK leukemic cells

**Figure 2:**
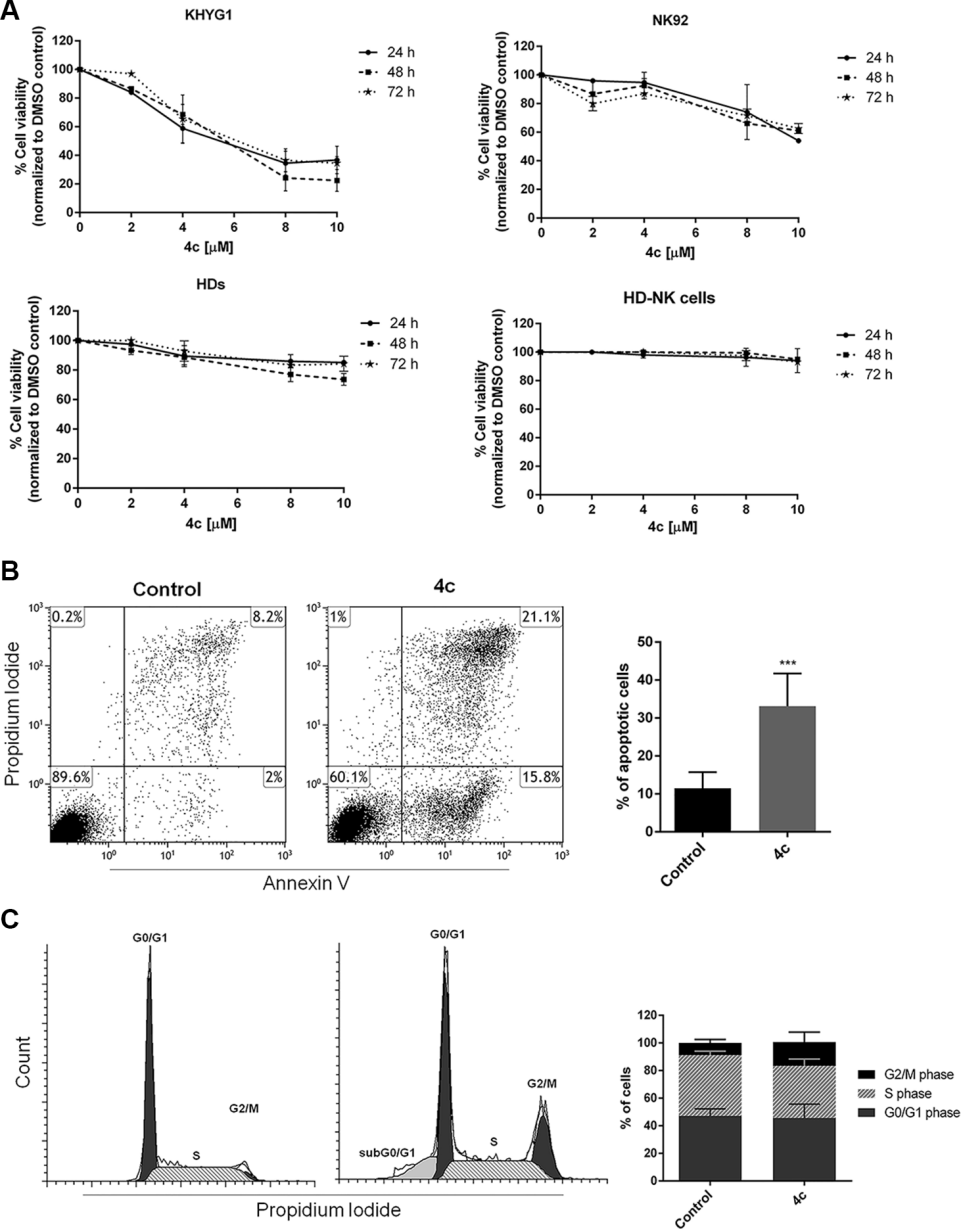
4c compound reduced cell viability inducing apoptosis and cell cycle arrest in NK leukemic cells (**A**) Viability of KHYG1, NK92, 3 PBMCs from HDs (HDs) and NK primary cells sorted from 3 HDs (HD-NK cells) was evaluated by MTS assay after treatment with 4c compound at different concentration (2–10 μM) for 24–72 hours. Results are expressed as percent of cell viability normalized to DMSO-treated control cells. The bar-graphs represent mean with S.D. from three independent experiments. (**B**) Apoptosis and (**C**) cell cycle analysis were evaluated by flow cytometer in NK cell line after 4c or DMSO vehicle treatment at 4 μM for 24 hours. Dot plots and cell cycle histograms show a single representative experiment, the bar-graphs represent mean with S.D. from three independent experiments. *P*-value is indicated by asterisk: ***p < 0.001.

We treated two cell lines (KHYG1 and NK92), 3 PBMCs from HDs and NK cells isolated from 3 HDs with 4c pyrazolo[3,4-*d*]pyrimidine compound or with dimethyl sulfoxide (DMSO) vehicle control at different concentrations (2–10 μM) for 24, 48 and 72 hours, cultured with IL2. After 4c treatment, we performed viability test which showed that 4c compound reduced viability of both cell lines in a dose-dependent manner. The effect was observed at 24 hours and remained constant in the other time points (Figure 2A). Importantly, both cell lines needed a significantly lower drug concentration (*p* < 0.01) to reach 50% reduction of viability (EC_50_) (Table 1). Interestingly, 4c compound had negligible effect in PBMCs and in purified NK cells from HDs (Figure 2A). Same results were obtained in primary NK cells from HDs treated with 4c compound and cultured without IL2 (Supplementary Figure S1).

**Table 1:** EC_50_ obtained in two cell lines and PBMCs and in purified NK cells from HD samples after 4c compound treatment (*P* value is < 0.01 for both cell lines vs HDs) (n.c.: not calculable)

	EC_50_ (μM)
KHYG1	5.4
NK92	10.6
HDs	59
HD-NK cells	n.c.

To further investigate cell death mechanism induced after treatment, we performed apoptosis and cell cycle analysis on KHYG1 cell line by cytometric analysis of Annexin V/propidium iodide (PI) and PI, respectively. After treatment with 4c compound at 4 μM for 24 hours, we observed a significant increase of apoptotic cells (*p* < 0.001) and cell cycle arrest in G2/M phase in treated KHYG1 respect to their control (Figure 2B–2C).

### Fyn phosphorylation is reduced after 4c compound treatment and it decreased Akt and P70 S6 kinase activation

**Figure 3:**
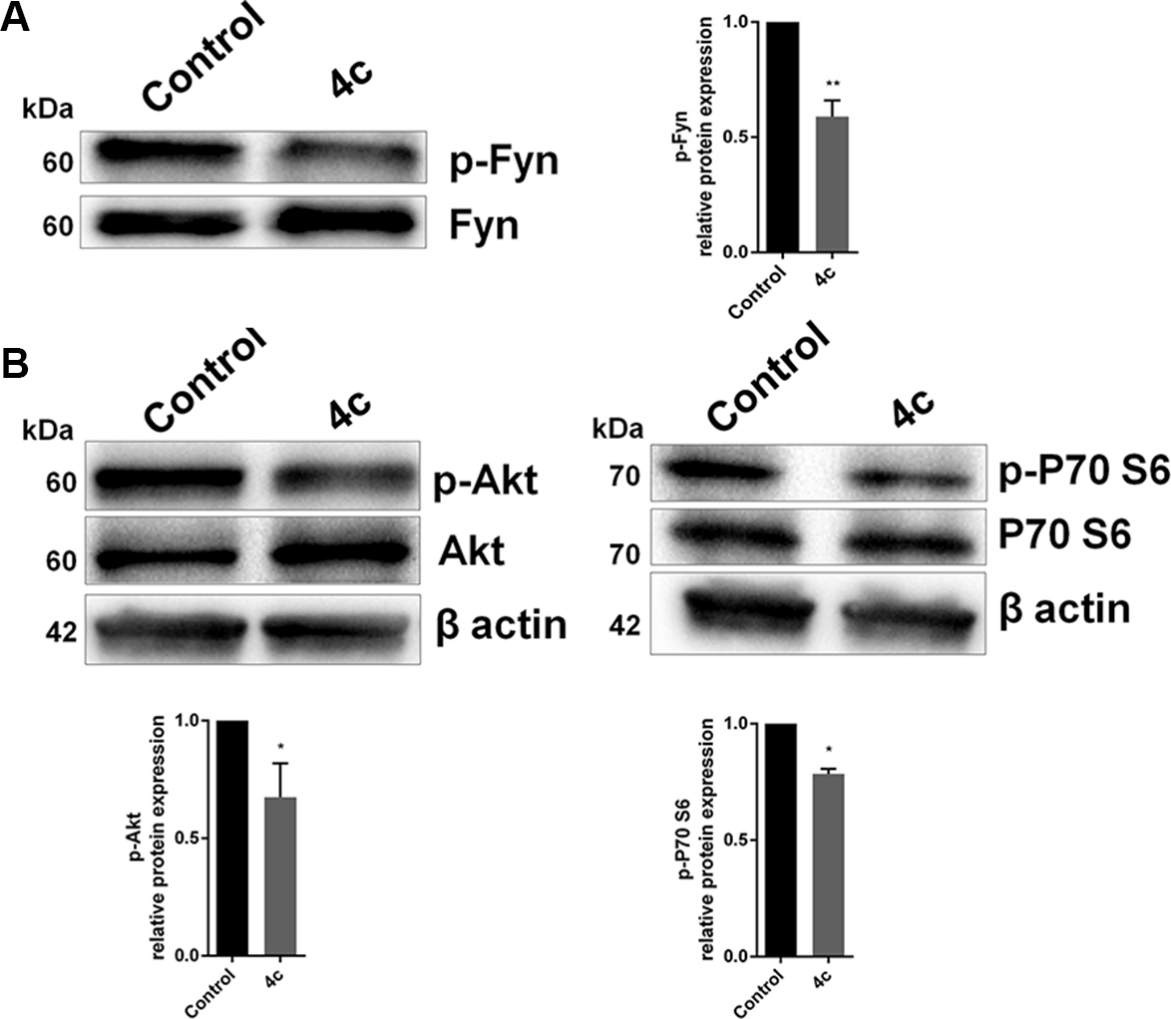
Inhibition on Fyn phosphorylation by 4c compound decreased Akt and P70 phosphorylation WB analysis of (**A**) phospho-Fyn and (**B**) total and phosphorylated Akt and P70 S6 kinase in NK cell line treated with 4c compound compared to control. Protein levels were normalized on β-actin signals. Bar-graphs represent mean with S.D. from three independent experiments. *P*-values are indicated by asterisk: *p < 0.05, **p < 0.01.

To verify Fyn inhibition we performed its immunoprecipitation in KHYG1 cell line treated with 4c compound or with DMSO vehicle control and we detected its phosphorylation. We observed that Fyn phosphorylation significantly decreased after treatment (*p* < 0.01; Figure 3A).

We also explored, by WB, the activation of two protein involved in Fyn pathway, Akt and P70 S6 kinase. Our data showed that there was a decrease of phosphorylation of Akt and P70 S6 kinase after treatment with 4c compound (*p* < 0.05; Figure 3B).

### Gene expression and protein profile of treated NK leukemic cells showed the activation of apoptotic pathways

**Figure 4:**
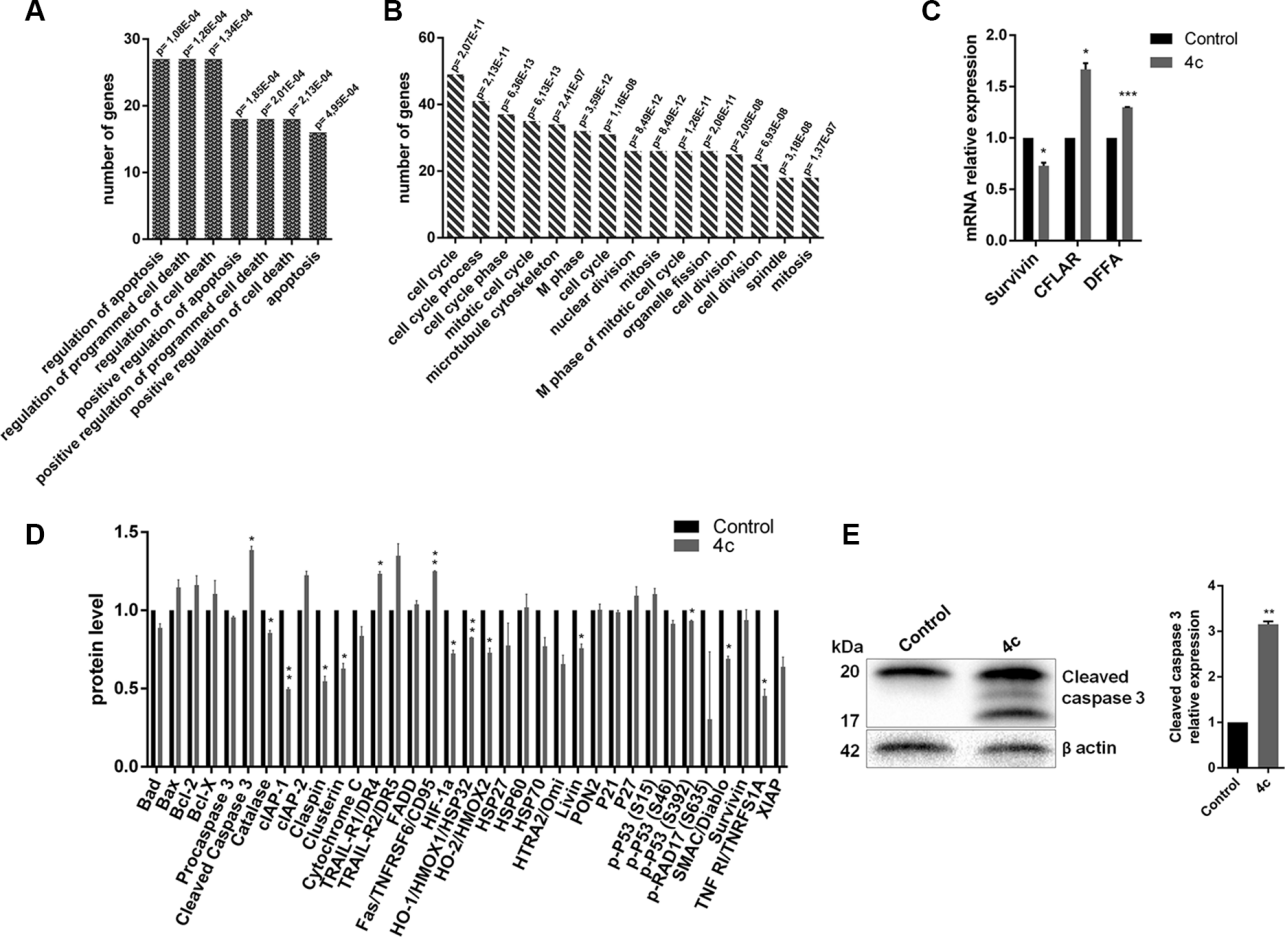
Activation of apoptotic pathways in 4c treated cell line (**A**–**B**) Bar-graphs of the first cluster up-regulated (A) and down-regulated (B) genes resulting by DAVID bioinformatic tool in GEP analysis of KHYG1 treated with 4c compound or with DMSO control. (**C**) qRT-PCR of *Survivin*, *CFLAR* and *DFFA* in KHYG1 treated vs control. Bar-graphs represent mean with S.D. from three independent experiments. (**D**) Bar-graphs of protein levels resulted from apoptotic protein array and (**E**) WB analysis of caspase 3 in KHYG1 treated vs control. *P*-values are indicated by asterisk: *p < 0.05, **p < 0.01, ***p < 0.001.

We performed gene expression profile (GEP) analysis of KHYG1 cell line after 4 μM drug incubation at 24 hours compared to control. A total of 697 genes (297 up-regulated vs 400 down-regulated genes) were differentially expressed. Ingenuity Pathway Analysis (IPA) gene ontology analysis demonstrated that most of genes were involved in cellular growth, death, development and cell cycle (Table 2). Moreover, analysis carried out with DAVID let us to cluster all genes in the same functions indicated by IPA. In fact, the first up-regulated cluster genes [e.g. *BCL2-like 13, Caspase-Related Inducer of Apoptosis* (*CFLAR*), *DNA Fragmentation Factor Alpha* (*DFFA*) and *Fas Ligand*] included pro-apoptotic functions (Figure 4A and Supplementary Material, Table S1); conversely, the first down-regulated cluster genes [e.g. *Survivin*, *Cell Division Cycle 34*, *Aurora Kinase A*] included pro-survival functions such as cell cycle progression, cell division, mitosis (Figure 4B and Supplementary Material, Table S2).

**Table 2:** Molecular and cellular functions of up- and down-regulated genes resulting from IPA gene ontology analysis in GEP analysis of 4c compound vs DMSO treated KHYG1 cells

Molecular and Cellular Functions	*p*-value range	Molecules
Cellular Growth and Proliferation	7.29E-04/5.16E-15	259
Cellular Function and Maintenance	6.79E-04/5.82E-14	126
Cell Death and Survival	7.81E-04/6.66E-14	239
Cellular Development	5.67E-04/6.26E-13	227
Cell Cycle	7.64E-04/2.48E-11	126

We validated GEP data by qRT-PCR confirming the down-regulation of anti-apoptotic gene, *Survivin* (*p* < 0.05), and the up-regulation of two pro-apoptotic genes, CFLAR (*p* < 0.05) and *DFFA* (*p* < 0.001), in KHYG1 treated vs control (Figure 4C).

Subsequently, to corroborate apoptosis and GEP data, we also performed an array analysis of multiple apoptotic proteins after treatment. As expected, we observed a low expression of anti-apoptotic proteins as cellular Inhibitors of Apoptosis Protein 1 (cIAP1), Claspin, Clusterin, Heat Shock Protein32 (HSP32), Livin, and high expression of apoptotic ones as cleaved caspase 3, TRAIL receptor 2 (TRAIL-R2) and Fas (Figure 4D).

In order to validate protein array, we showed a significantly increase of cleaved caspase 3 level (***p*** < 0.05) in 4c compound treated cell line by WB (Figure 4E).

### 4c pyrazolo[3,4-*d*]pyrimidine compound induced cytotoxic effect and cell cycle arrest in primary NK leukemic cells

**Figure 5:**
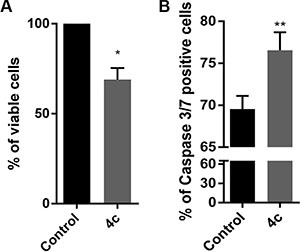
4c treatment reduced viability and induced caspase 3-mediated apoptosis in PBMC from CLPD patients (**A**) Trypan blue count was performed in primary cells, isolated from 3 HDs and 3 CLPD patients (UNP: 6–8) and (**B**) Caspase 3/7 activity assay on 3 CLPD patients (UNP: 6–8) after 24 hours of treatment with 4c compound at 4 μM concentration. *P*-values are indicated by asterisk: *p < 0.05, **p < 0.01.

In order to evaluate Fyn inhibitor effects on primary cells, we exposed PBMCs from CLPD patients to 4 μM of 4c compound for 24 hours and we evaluated cell viability by trypan blue count. We observed a decrease of 30% of viable cells in treated PBMCs respect control (Figure 5A). Notably, in treated NK cells from leukemic patients there was an increase of active caspase 3/7 level indicating an apoptosis caspase 3-mediated (*p* < 0.01, Figure 5B).

**Figure 6:**
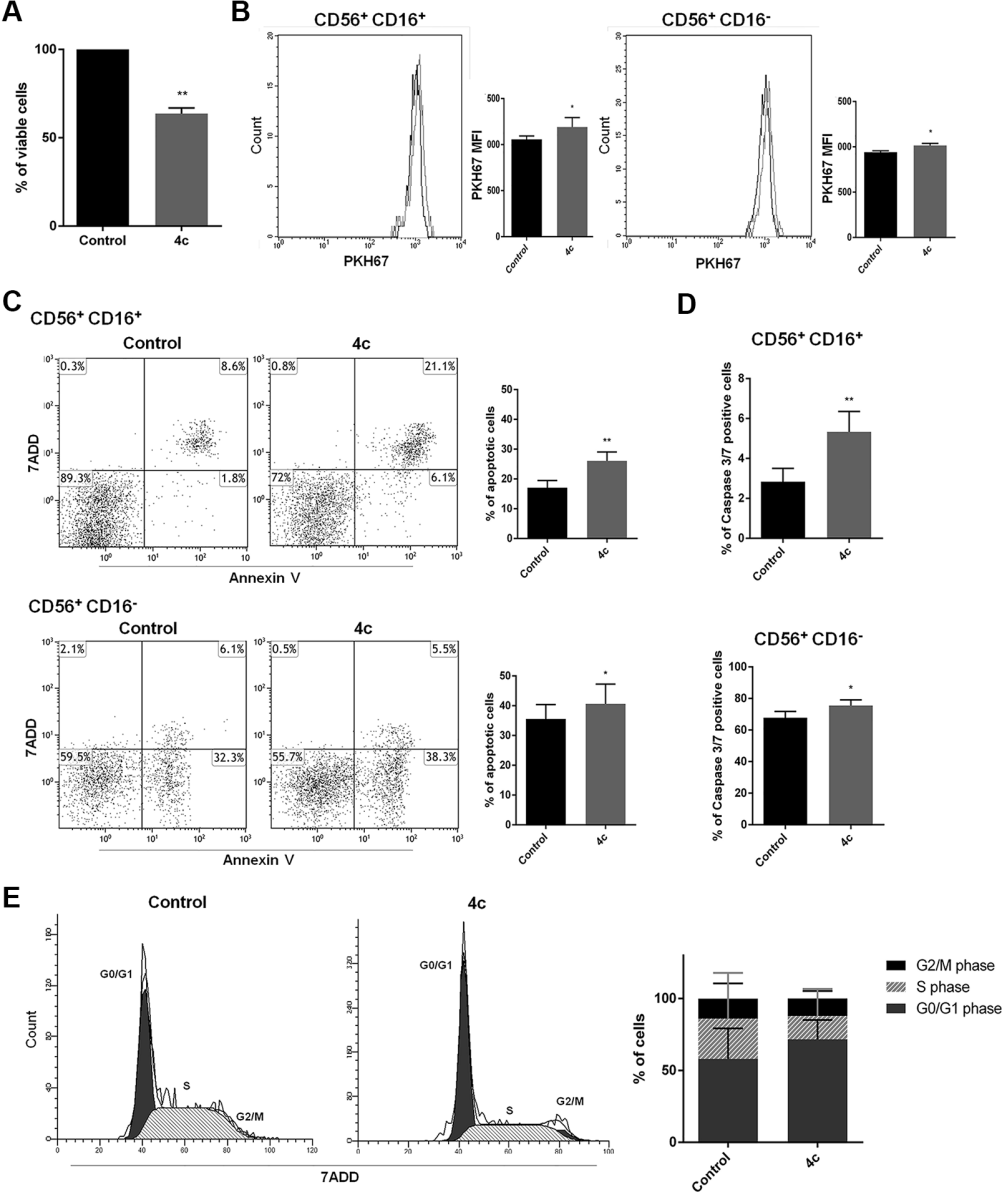
4c compound treatment reduced cell viability and proliferation, induced caspase 3-mediated apoptosis and cell cycle arrest in primary NK leukemic cells On 4c treated or not primary NK cells sorted from PBMCs of 3 NK-CLPD patients (UPN: 6–8) (**A**) Trypan blue count, (**B**) PKH67 proliferation test, (**C**) Apoptosis and (**D**) Caspase 3/7 activity assay, (**E**) Cell cycle analysis were performed. Proliferation, apoptosis and caspase 3/7 level were analyzed on CD56^+^/CD16^+^ and CD56^+^/CD16^−^ NK cell populations. *P*-values are indicated by asterisk: *p < 0.05, **p < 0.01. (MFI= mean fluorescent intensity).

To better elucidate 4c effects on primary NK cells, we sorted CD56^+^ NK cells from 3 PBMCs of CLPD patients and we exposed them to 4c compound for 24 hours. After incubation time, we evaluated viability by trypan blue count, proliferation by PKH67 labeling, apoptosis and cell cycle by Annexin V/7-Amino-Actinomycin D (7ADD) and PI respectively. Firstly, we observed a decrease of 37% of viable cells (Figure 6A). Furthermore, we analyzed proliferation, apoptosis and caspase 3/7 rate both on CD56^+^/CD16^+^ and CD56^+^/CD16^−^ after 4c treatment. We noted a reduction of proliferation in both CD56^+^/CD16^+^ and CD56^+^/CD16^−^cells (Figure 6B). Moreover, in both treated populations, we observed a significantly increased apoptosis compared to their respective control (+ 17% in CD56^+^/CD16^+^ cells and + 5.4% in CD56^+^/CD16^−^ cells; *p* < 0.01 and *p* < 0.05 respectively) (Figure 6C). The 4c effect on apoptosis was also confirmed by increased expression of caspase3/7 levels in both population (+ 2.5% in CD56^+^/CD16^+^ cells and + 8% in CD56^+^/CD16^−^ cells; *p* < 0.01 and *p* < 0.05 respectively) (Figure 6D). Cell cycle analysis after 4c treatment showed that there was an increase of number of NK primary cells in G0/G1 phase (Figure 6E).

### 4c compound treatment induced phenotype changes in NK primary cells

**Figure 7:**
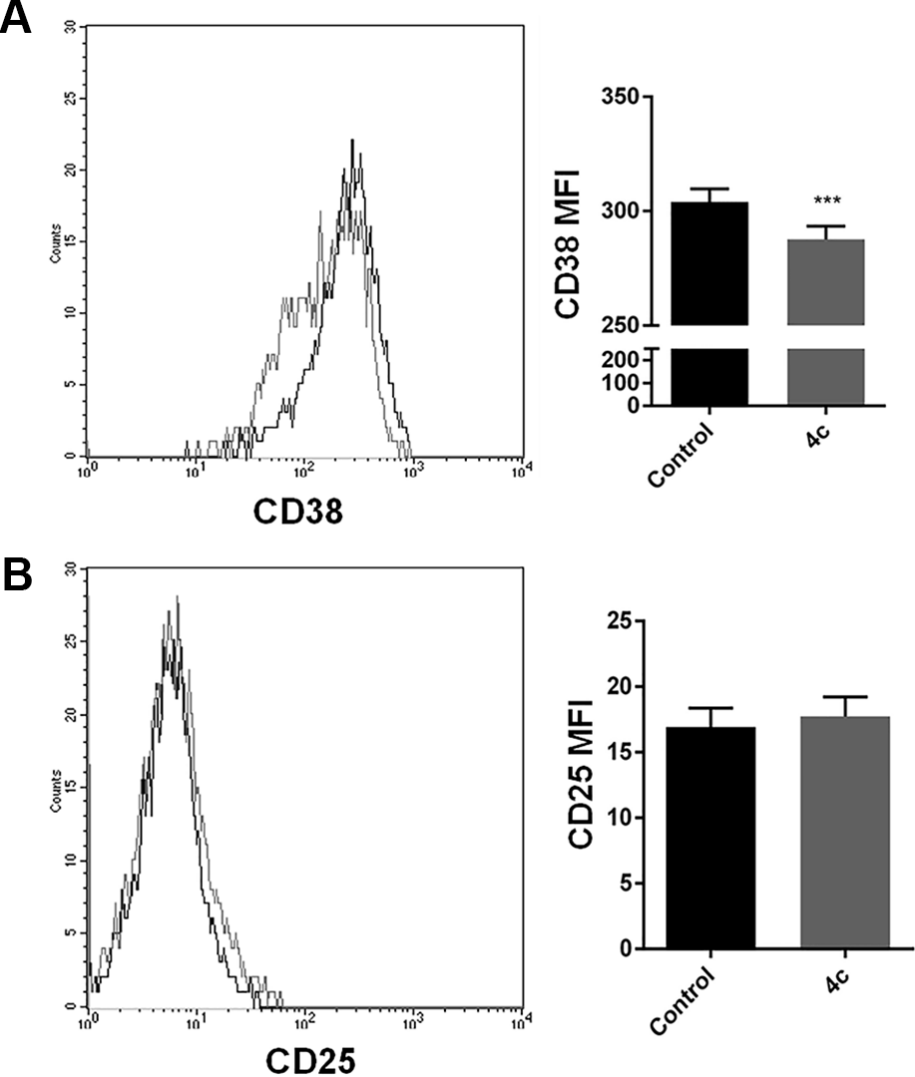
4c compound treatment induced phenotype changes in NK primary cells On 4c primary NK cells sorted from PBMCs of 3 CLPD patients (UPN: 6–8) (**A**) CD38 and (**B**) CD25 expression were evaluated after 4c treatment. *P*-values is indicated by asterisk: ***p < 0.001. (MFI = mean fluorescent intensity)

To evaluate if 4c compound influence NK cell activation, we sorted CD56^+^ NK cells from 3 PBMCs of CLPD patients and we exposed them to 4c compound for 24 hours to study cell phenotype changes. We analyzed the expression of two markers related to NK activation, CD38 and CD25. We observed that there was a decrease of CD38 expression and similar expression of CD25 after treatment respect to control in NK cells (Figure 7A–7B).

## DISCUSSION

NK cell-type large granular lymphocyte leukemia is a rare neoplasm divided in two subtype which could be considered different and independent neoplasms because they have distinct clinical course and management. Chronic lymphoproliferative subtype is characterized by indolent clinical course lacking effective therapeutic approaches [18]; the number of circulating NK cells remains stable for long periods of time and some cases have been reported to show spontaneous regression. On the other hand, aggressive NK leukemia subtype is a fulminant disorder which is not curable.

By tissue-specific pattern of mRNA [16], it was identified Fyn like as a gene more expressed in normal NK cells respect to other tissues. It is required to control cytotoxicity and cytokine production in NK cells [19, 20], and it has a critical role in NK and T-cell development and activation [9, 21].

Since NK leukemia is characterized by an outgrowth of CD3^−^ CD16/56^+^ cells, Fyn could be a possible target in this hematological disease. In fact, analyzing its expression in NK leukemic cells, we found an increased Fyn transcript level in PBMCs from CLPD patients respect to HDs. Interestingly, Fyn protein levels was higher in CLPD patients and in NK cell lines respect to HDs. In these last samples Fyn protein level was not detectable, perhaps for the lower percent of NK cells in HD-PBMCs (< 10%) respect to CLPD patients (20–40%), to equal protein loaded lysate in western blotting assay. The over-expression of Fyn is documented in different cancers, such as CML, brain, prostate and breast tumors where it facilitates the growth, the migration of tumor cells and genomic instability [22–26]. Recently, *FYN* mutations were found in peripheral T-cell lymphomas where they impaired DNA damage response and escape from immune surveillance [14].

Our previous data showed that 4c pyrazolo[3,4-*d*]pyrimidine compound is able to inhibit Fyn kinase in a cell-free assay and to induce apoptosis and cell cycle arrest in CML cell line [17].

In this paper, for the first time, we investigated the effect of this compound on NK cell leukemia.

First of all, we demonstrated that 4c reduced cell viability in NK cell lines. Interestingly, it had no effect on both PBMCs and NK purified cells from HDs.

Since it was discovered previously that exposure to stimulatory factors such as the cytokine IL2 enhanced NK cell potency significantly [27], we cultured primary HD-NK cells with and without IL2 in order to evaluate if 4c compound could have diverse effect on NK cells differently activated. We observed that 4c compound had no effect on HD-NK cell viability in both culture conditions. So the presence of IL2 not influenced response to 4c treatment.

These results allowed us to conclude that 4c compound acted only on NK leukemic cells and not on healthy NK cells. Moreover, it not influenced viability of other PBMC population.

We obtained different percent of viability reduction in cell lines after 4c treatment. More precisely, the major effect was observed in KHYG1 respect to NK92. The diverse response to 4c compound of the two cell lines could be due to their different origin. In particular, KHYG1 was established from peripheral blood of a woman with aggressive NK leukemia at diagnosis, while NK92 was established from a man with non-Hodgkin's lymphoma with large granular lymphocytes, more corresponding to chronic subtype.

We decided to study in deep the viability reduction in NK aggressive KHYG1 cells and demonstrated that 4c induced a G2/M cell cycle arrest and a significant apoptosis.

Recent studies show the possibility to conduct NK malignant cells to apoptosis thus supporting a possible therapeutic option. Quoc Trung L *et al*. showed that resveratrol induced G0/G1 cell cycle arrest and apoptosis in NK leukemic cell lines by inhibiting STAT3 signaling [28]; similar results were obtained by Sun X *et al*. using a combination of drugs, vorinostat and cladribine [29].

To demonstrate that effects on cell viability were due to Fyn inhibition, we confirmed its reduced phosphorylation. These findings are in agreement with Ninio-Many L *et al*. who revealed that miR-125a-3p causes an arrest of cell cycle at G2/M stage and a decreases cell viability in a Fyn-dependent manner [30]. Other studies, in glioma and prostate cells, revealed similar results after Fyn down modulation [23, 31]. All this confirms the fact that Fyn is considered an important regulator of cell cycle, growth and proliferation [32–34].

To the best our knowledge, this is the first study concerning the Fyn targeting in NK malignancies.

Moreover, we investigated Fyn downstream pathway. Since some studies have shown that Src kinases, including Fyn, may be required for Akt activation by a tyrosine phosphorylation-dependent mechanism [35, 36], we also investigated the Akt behavior after 4c treatment and we observed that Fyn inhibition reduces Akt phosphorylation. Additionally, our data showed a reduction of P70 S6 kinase phosphorylation, an important downstream target of Akt involved in cell survival and in apoptosis inhibition [37, 38].

Furthermore, GEP analysis of 4c treated KHYG1 cells confirmed the down-regulation of pro-survival gene pathways and the up-regulation of apoptotic functions. In particular, we validated by qRT-PCR the reduction of *Survivin*, and the increase of *CFLAR* and *DFFA* mRNA. Survivin, a member of the inhibitor of apoptosis protein (IAP) family, regulates leukemic NK cell survival via ERK/MAPK signaling [39]. Interestingly, Liu *et al*. demonstrated that targeting of survivin may be a possible therapeutic approach for NK leukemia because it is highly expressed in both aggressive and chronic leukemic NK cells respect to normal ones [40].

The first up-regulated gene, *CFLAR*, also known as Casper/cFLIP, is a FADD- and Caspase-Related inducer of apoptosis and its over-expression induces apoptotic cell death [41–43]. It also play a critical role in autophagy, necroptosis and apoptosis in T-lymphocytes [44] and it has prognostic and therapeutic relevance in acute myeloid leukemia (AML) [45] as well as in development of drug resistance [46]. *DFFA* gene is the substrate for caspase 3 and triggers DNA fragmentation during apoptosis [47, 48].

In addition, the protein array confirmed the down modulation of survivin, of other members of IAP family, such as cIAP-1, XIAP, livin [49, 50] and of cell cycle regulators such as Cluspin [51], and the high modulation of pro-apoptotic ones, such as cleaved caspase 3.

Interestingly, dot blot assay showed a decrease of Cytochrome C and Smac/DIABLO and an increase of Fas levels suggesting the activation of extrinsic apoptotic pathway [52]. We also confirmed, by western blotting (WB), the presence of cleaved caspase 3 after treatment letting us to assert that 4c Fyn inhibitor compound is able to induce apoptotic death caspase 3-mediated in NK malignant cells.

Finally, we demonstrated that 4c pyrazolo[3,4-*d*]pyrimidine compound reduced viability and induced apoptosis in PBMCs and in NK cells from patients with NK leukemia.

In human PBMCs the NK cells can be divided on the basis of the expression of CD56 and CD16 markers. CD56^+^/CD16^+^ and CD56^+^/CD16^−^ populations show different proliferation, redox state and function. Specifically, CD56^+^/CD16^−^ cells are responsible for immunoregulatory cytokines production, CD56^+^/CD16^+^ cells are responsible for the cytotoxic lysis of target cells [53–54]. On the basis of these considerations, we analyzed the 4c effects on both NK purified population. We demonstrated that there was a reduction of viability after treatment but also a reduction of proliferation and an increase of apoptosis caspase dependent in both population CD56^+^/CD16^+^ and CD56^+^/CD16^−^. From cell cycle analysis, 4c compound resulted to be cytostatic, in fact there was an increase of G0/G1 phase.

In order to study the 4c effect on NK cell phenotype, we evaluated two important surface molecule involved in NK cells activation, such as CD38 and CD25 [55]. We demonstrated that 4c treatment reduced the expression of CD38, while CD25 expression remained constant. It means that NK treated cells could be slightly active.In summary, the present study demonstrated that Fyn kinase has a role in the pathogenesis of NK leukemia. Moreover, inhibition of Fyn phosphorylation by 4c pyrazolo[3,4-*d*]pyrimidine compound induces apoptosis and cell cycle arrest in NK leukemic cells.

Fyn targeting by 4c compound could represent a potential and possible strategy for NK leukemia treatment.

## MATERIALS AND METHODS

### Patients, cell lines and chemical

Peripheral blood samples from HDs and NK-CLPD (patient characteristics are shown in Table 3) were obtained with informed consent. PBMCs were isolated by Ficoll-hypaque gradient separation and NK cells were isolated by cell sorter MoFlo Atrios (Beckman Coulter, Brea, California, USA) by labeling with anti-CD56 phycoerythrin (PE) (Becton Dickinson, BD, Franklin, NJ, USA). Human NK cell lines, KHYG1 and NK92 [56, 57], were purchased from Leibniz-Institut DSMZ – Deutsche Sammlung von Mikroorganismen und Zellkulturen GmbH and American Type Culture Collection, respectively. NK primary cells and cell lines were cultured in RPMI 1640 (Gibco, Life technologies, Carlsbad, CA, USA) supplemented with 10% fetal bovine serum (FBS, Gibco), 1% of penicillin-streptomycin (Gibco) and 10 ng/ml interleukin-2 (IL-2, Miltenyi Biotec, Auburn, CA) at 37°C and 5% CO_2_.

**Table 3:** Clinical characteristics of NK-CLPD patients

UPN	Gender	Age	NK cells
1	F	65	> 60%
2	M	63	> 60%
3	M	49	40–60%
4	M	49	40–60%
5	M	45	40–60%
6	F	77	30–40%
7	M	45	30–40%
8	M	51	20–30%

Abbreviations: UPN, unique patient number; F, female; M, male.

4c pyrazolo[3,4-*d*]pyrimidine compound, given by Lead Discovery Siena s.r.l. (patent: WO2016066755), was dissolved in DMSO (Sigma Aldrich, St Louis, MO, USA) and diluted in FBS for cell treatments.

### RNA extraction and qRT-PCR

Total RNA was extracted with AllPrep DNA/RNA/miRNA Universal Kit (Qiagen GmbH, Hilden, Germany) according to the manufacturer's instructions. Reverse transcription was performed using Transcriptor First Strand cDNA Synthesis kit (Roche, Indianapolis, IN, USA).

mRNA expression was evaluated by qRT-PCR, performed on Light Cycler 480 II (Roche) with intercalating dye SYBR Green I Master Mix (Roche) using 100 ng cDNA at the following conditions: 95°C for 10 min, 45 cycles at 95°C for 10 sec, 60°C for 10 sec, 72°C for 15 sec. Each sample was analyzed in triplicate. Relative mRNA expression values were normalized using GAPDH as reference gene and Universal Human Reference RNA (Qiagen) as calibrator and calculated on the basis of the E^-ΔΔCp^ method.

The primer sequences of human *Fyn*, *GAPDH*, *Survivin*, *CFLAR* and *DFFA* were as follows: Fyn forward 5′-AGATTGCTGACTTCGGATTG-3′, Fyn reverse 5′-CAGACTTGATTGTGAACCTC-3′, GAPDH forward 5′-AGGCTGAGAACGGGAAGC-3′, GAPDH reverse 5′-CCATGGTGGTGAAGACGC-3′, Survivin forward 5′-AGAACTGGCCCTTCTTGGAGG-3′, Survivin reverse 5′-CTTTTTATGTTCCTCTATGGGGTC-3′, CFLAR forward 5′-TGGTAAGGCATGCTTCCAGA-3′, CFLAR reverse 5′-ACAGTATCAGAAGGTGGGGC-3′, DFFA forward 5′-CACTCCAACAGGTGCTTGAC-3′, DFFA reverse 5′-AGTGCAGTAAGGATGTGGCT-3′.

### Gene expression profile analysis

Total RNA was quantified with a NanoDrop 2000c spectrophotometer (Thermo Scientific, Wilmington, DE, USA) and its quality was assessed by capillary electrophoresis on an Agilent 2100 Bioanalyzer (Agilent Technologies, Inc, Santa Clara, CA) using RNA 6000 Nano Assay Kit (Agilent). Only samples with RNA integrity number (RIN) > 7 were used.

Samples preparation, hybridization, staining and scanning of Illumina HumanHT12 v4.0 Expression BeadChip array (Illumina Inc., San Diego, CA, USA) on HiScanSQ system (Illumina Inc.) was performed as described before [58].

The intensity files were loaded into the Illumina Genome Studio software for quality control and gene expression analysis. Quantile normalization algorithm was applied on the data set to correct systematic errors, values below a detection score of 0.05 were filtered out and missing values were imputed. Differently expressed genes (DEGs) were selected with differential score (DiffScore) cutoff set at ± 13 (*p* < 0.05). Microarray data were submitted to Array Express under accession number E-MTAB-4536. DEGs list was used to evaluate the functional behavior in terms of Biological Processes performing an enrichment analysis with IPA (Ingenuity Systems; Mountain View, CA, USA). DEGs list was uploaded also into DAVID bioinformatic tool [59] and functional annotation clustering was evaluated separately for up- and down-regulated genes.

### Western blotting analysis

Cells were lysed as previously reported [60]. 80 μg was subjected to sodium dodecyl sulfate polyacylamide gel electrophoresis on a 10% gel under reducing conditions and then electrotransferred onto a polyvinylidene difluoride membranes using Trans Blot Turbo Transfer System (BioRad, Hercules, CA, USA). Membranes were probed with primary antibodies directed against Fyn, phospho-Src Family (Tyr416), phospsho-AKT (Ser473), Akt, phospho-P70 S6 kinase (Thr389), P70 S6 kinase, β actin (Cell Signaling, Beverly, MA, USA), active caspase 3 (Abcam, Cambridge, UK) and then incubated with secondary antibody (horseradish peroxidise-conjugated goat anti-mouse or anti-rabbit; Cell Signaling). Immune complexes were detected by ECL chemiluminescence system (Bio-Rad Laboratories), as recommended by the manufacturer. Densiometric analysis was performed using BioRad Image Lab 4.1 software. The intensity of bands of all proteins was normalized to the β actin signal.

### Immunoprecipitation

Protein immunoprecipitation was carried out starting from 1 mg of total protein extracts. Lysates were pre-cleared by incubating with protein A/G-Agarose (Santa Cruz Biotechnology) for 1 hour, subsequently incubated with anti-Fyn antibody for 18 hours and then with fresh A/G-Agarose for 2 hours. All incubation were conducted at 4°C. The product was collected by centrifugation and washed twice. WB analysis was performed as previously reported.

### Proteome profiler array

Protein extract of KHYG1 cell line, treated with 4c compound at 4 μM or with DMSO vehicle control for 24 hours, were subjected to the human apoptosis array following the manufacturer's instructions (Human Apoptosis Array kit, R&D Systems, Abingdon, UK).

### Cell viability

KHYG1 and NK92 cell lines, PBMCs and NK cells from HDs were seeded into 96-well plates (3 × 10^4^ cells/100 μl) and incubated with 4c compound at increasing concentrations (2–10 μM) for 24, 48 and 72 hours. Cells treated with DMSO vehicle were used as control. Cell viability was determined using the CellTiter 96 Aqueous One Solution assay kit (MTS, Promega, Madison, WI, USA). The optical density was measured at 492 nm. Cellular viability was calculated as percentage of viable cells compared with control. All experiments were conducted in triplicate. EC_50_ values were obtain by GraphPad Prism (GraphPad Prism, San Diego, CA). Viability of cell lines and primary NK cells from NK-CLPD was assessed by Trypan blue dye count method.

### Functional tests

KHYG1 and NK cells from NK-CLPD samples were treated with 4 μM of 4c compound or with DMSO vehicle control for 24 hours (cell density 3 × 10^5^ cells/ml) and used in:

### Apoptosis assay

Apoptosis of KHYG1 was evaluated by cytometric analysis of Annexin V and Propidium Iodide (PI)-stained cells using fluorescein isothiocyanate (FITC) Annexin V Apoptosis Detection kit I (BD). NK cells isolated from NK-CLPD samples were firstly labeled with anti CD56 PE and CD16 allophycocyanin (APC), than labeled with Annexin V and 7-Amino-Actinomycin D (7ADD, BD) to evaluate apoptosis. Stained cells were acquired using FACSCalibur flow cytometer and analyzed by CellQuest Pro software (BD). Single positive for Annexin V and double positive for Annexin V and PI/7ADD cells were interpreted as signs of early and late phases of apoptosis respectively.

### Cell cycle analysis

After treatment KHYG1 cells were fixed in cold ethanol 70% for 1 hour, then labeled with PI/RNase staining solution for 30 minutes. Instead, after treatment, NK cells from NK-CLPD samples were labeled with anti-CD56 PE and anti-CD16 APC, fixed and permeabilzed by Intracell kit (Immunostep, S.L. Avda), then labeled with 7ADD/RNase staining solution for 30 minutes. Samples were acquired by FACSCalibur (BD). Data were analyzed by ModFit LT Software (Verity Software House).

### Caspase 3/7 activity assay

NK primary cells, treated with 4c compound or with DMSO vehicle for 24 hours (cell density 1 ^×^ 10^6^/ml), were labeled with anti-CD56 PE and anti-CD16 APC, then incubated with 1 μM of CellEvent caspase3/7 Green Detection Reagent (Life technologies, Carlsbad, CA, USA) in complete medium at 37°C for 30 minutes as manufacturer's protocol. Stained cells were analyzed by FACSCalibur cytometer (BD).

### Proliferation assay

NK primary cells were stained with PKH67 (PKH67 Green Fluorescent Cell Linker Kit for General Cell Membrane Labeling, Sigma Aldrich) as manufacturer's protocol, treated with 4c compound or with DMSO vehicle control for 24 hours and analyzed by FACSCalibur cytometer (BD).

### Analysis of NK phenotype

NK primary cells, treated with 4c compound or with DMSO vehicle control for 24 hours, were labeled with anti-CD25 FITC, anti CD38-APC (BD) and analyzed by FACSCalibur cytometer (BD).

### Statistical analysis

Statistical significance was determined using a paired or unpaired t test by GraphPad Prism. All error bars represent the standard deviation (SD) of the mean.

## SUPPLEMENTARY MATERIALS FIGURE AND TABLES




