# Supplementary material for: A Pyrazolo[3,4-d]pyrimidine compound inhibits Fyn phosphorylation and induces apoptosis in natural killer cell leukemia
Source: Oncotarget. 2016 Aug 22;7(40):65171–84. doi: 10.18632/oncotarget.11496 (PMC5323146; doi:10.18632/oncotarget.11496)
Supplement: Supplementary file 1 [file oncotarget-07-65171-s001.pdf]

A Pyrazolo[3,4-*d*]pyrimidine compound inhibits Fyn phosphorylation and induces apoptosis in natural killer cell Leukemia

Supplementary Materials

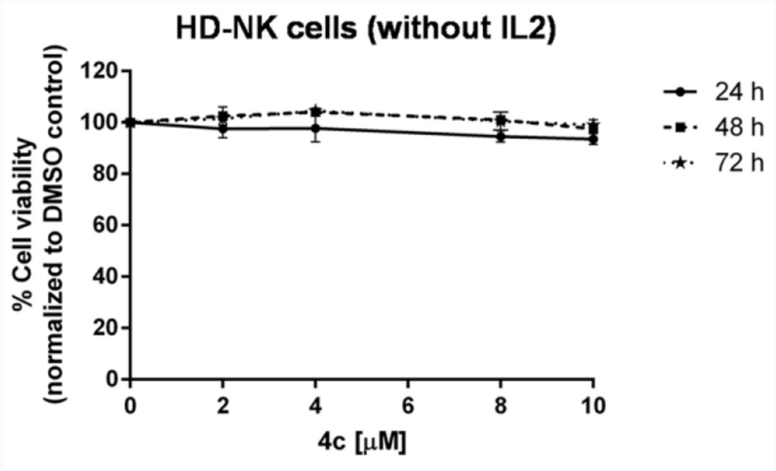

Supplementary Figure S1: Viability of 3 samples of NK primary cells sorted from HDs (HD-NK cells) cultured without IL2 was evaluated by MTS assay after treatment with 4c compound at different concentration (2–10 µM) for 24–72 hours. Results are expressed as percent of cell viability normalized to DMSO-treated control cells. The bar-graphs represent mean with S.D. from three independent experiments.

Supplementary Table S1: First cluster up-regulated genes resulting by DAVID bioinformatic tool in GEP analysis of KHYG1 treated with 4c compound or with DMSO control

Term	Count	*P*-value	Genes
GO:0042981~regulation of apoptosis	27	1.08E-04	TXNIP, CFLAR, GIMAP5, TM2D1, DFFA, RXRA, ITGA1, FASLG, RPS27L, IFI16, BCL2L13, STK4, SELS, ATM, TXNDC12, CDKN1A, FNTA, BTG1, SH3GLB1, BCL2, DAD1, CSTB, FAIM3, GLO1, CFDP1, MYC, PHLDA1
GO:0043067~regulation of programmed cell death	27	1.26E-04	TXNIP, CFLAR, GIMAP5, TM2D1, DFFA, RXRA, ITGA1, FASLG, RPS27L, IFI16, BCL2L13, STK4, SELS, ATM, TXNDC12, CDKN1A, FNTA, BTG1, SH3GLB1, BCL2, DAD1, CSTB, FAIM3, GLO1, CFDP1, MYC, PHLDA1
GO:0010941~regulation of cell death	27	1.34E-04	TXNIP, CFLAR, GIMAP5, TM2D1, DFFA, RXRA, ITGA1, FASLG, RPS27L, IFI16, BCL2L13, STK4, SELS, ATM, TXNDC12, CDKN1A, FNTA, BTG1, SH3GLB1, BCL2, DAD1, CSTB, FAIM3, GLO1, CFDP1, MYC, PHLDA1
GO:0043065~positive regulation of apoptosis	18	1.85E-04	TXNIP, CFLAR, TM2D1, DFFA, RXRA, ITGA1, FASLG, RPS27L, IFI16, BCL2L13, STK4, ATM, TXNDC12, CDKN1A, SH3GLB1, BCL2, MYC, PHLDA1
GO:0043068~positive regulation of programmed cell death	18	2.01E-04	TXNIP, CFLAR, TM2D1, DFFA, RXRA, ITGA1, FASLG, RPS27L, IFI16, BCL2L13, STK4, ATM, TXNDC12, CDKN1A, SH3GLB1, BCL2, MYC, PHLDA1
GO:0010942~positive regulation of cell death	18	2.13E--04	TXNIP, CFLAR, TM2D1, DFFA, RXRA, ITGA1, FASLG, RPS27L, IFI16, BCL2L13, STK4, ATM, TXNDC12, CDKN1A, SH3GLB1, BCL2, MYC, PHLDA1
Apoptosis	16	4.95E-04	CFLAR, LITAF, TM2D1, DFFA, GZMA, TNFRSF12A, FASLG, BCL2L13, STK4, ATM, TXNDC12, SH3GLB1, BCL2, DAD1, SRGN, PHLDA1

Supplementary Table S2: First cluster down-regulated genes resulting by DAVID bioinformatic tool in GEP analysis of KHYG1 treated with 4c compound or with DMSO control. See Supplementary_ Table_S2.
