# Supplementary material for: A Pyrazolo[3,4-d]pyrimidine compound inhibits Fyn phosphorylation and induces apoptosis in natural killer cell leukemia
Source: Oncotarget. 2016 Aug 22;7(40):65171–84. doi: 10.18632/oncotarget.11496 (PMC5323146; doi:10.18632/oncotarget.11496)
Supplement: Supplementary file 2 [file oncotarget-07-65171-s002.docx]

**Table S2.** First cluster down-regulated genes resulting by DAVID bioinformatic tool in GEP analysis of KHYG1 treated with 4c compound or with DMSO control.

| **Term** | **Count** | **P-value** | **Genes** |
| --- | --- | --- | --- |
| GO:0000278~mitotic cell cycle | 35 | 6.13E-13 | PSMB10, PRC1, AURKA, CDC34, AURKB, GTSE1, SPC24, FAM83D, CDCA8, SAC3D1, CDKN2D, BUB1, PSMD2, CCNA2, TCF3, NUDC, CUL1, CDCA3, ANAPC5, DLGAP5, CCNF, CENPF, CDC20, ESPL1, BIRC5, PBK, DCTN1, NCAPD2, CDC25B, FSD1, CHMP1A, CCNB2, PSMD12, PLK1, AKAP8 |
| GO:0022403~cell cycle phase | 37 | 6.36E-13 | XRCC2, PRC1, AURKA, CDC34, AURKB, GTSE1, SPC24, FAM83D, RPA1, CDCA8, SAC3D1, CDKN2D, BUB1, H2AFX, CCNA2, TCF3, NUDC, CUL1, CDCA3, MKI67, ANAPC5, DLGAP5, CCNF, CENPF, CDC20, ESPL1, BIRC5, PBK, TACC3, DCTN1, NCAPD2, CDC25B, FSD1, CHMP1A, CCNB2, PLK1, AKAP8 |
| GO:0000279~M phase | 32 | 3.59E-12 | XRCC2, PRC1, AURKA, AURKB, SPC24, FAM83D, RPA1, CDCA8, SAC3D1, BUB1, H2AFX, CCNA2, NUDC, CDCA3, MKI67, ANAPC5, DLGAP5, CCNF, CENPF, ESPL1, BIRC5, CDC20, PBK, TACC3, DCTN1, NCAPD2, CDC25B, FSD1, CHMP1A, CCNB2, PLK1, AKAP8 |
| GO:0000280~nuclear division | 26 | 8.49E-12 | AURKA, AURKB, SPC24, FAM83D, CDCA8, SAC3D1, BUB1, CCNA2, NUDC, CDCA3, ANAPC5, DLGAP5, CCNF, CENPF, ESPL1, BIRC5, CDC20, PBK, DCTN1, CDC25B, NCAPD2, FSD1, CHMP1A, CCNB2, PLK1, AKAP8 |
| GO:0007067~mitosis | 26 | 8.49E-12 | AURKA, AURKB, SPC24, FAM83D, CDCA8, SAC3D1, BUB1, CCNA2, NUDC, CDCA3, ANAPC5, DLGAP5, CCNF, CENPF, ESPL1, BIRC5, CDC20, PBK, DCTN1, CDC25B, NCAPD2, FSD1, CHMP1A, CCNB2, PLK1, AKAP8 |
| GO:0000087~M phase of mitotic cell cycle | 26 | 1.26E-11 | AURKA, AURKB, SPC24, FAM83D, CDCA8, SAC3D1, BUB1, CCNA2, NUDC, CDCA3, ANAPC5, DLGAP5, CCNF, CENPF, ESPL1, BIRC5, CDC20, PBK, DCTN1, CDC25B, NCAPD2, FSD1, CHMP1A, CCNB2, PLK1, AKAP8 |
| GO:0048285~organelle fission | 26 | 2.06E-11 | AURKA, AURKB, SPC24, FAM83D, CDCA8, SAC3D1, BUB1, CCNA2, NUDC, CDCA3, ANAPC5, DLGAP5, CCNF, CENPF, ESPL1, BIRC5, CDC20, PBK, DCTN1, CDC25B, NCAPD2, FSD1, CHMP1A, CCNB2, PLK1, AKAP8 |
| GO:0007049~cell cycle | 49 | 2.07E-11 | PSMB10, E2F2, LZTS1, XRCC2, PRC1, FOXM1, AURKA, CDC34, AURKB, GTSE1, CDT1, SPC24, FAM83D, RPA1, CDCA8, SAC3D1, CDKN2D, BUB1, PSMD2, H2AFX, SUPT5H, TCF3, CCNA2, NUDC, CUL1, CDCA3, MKI67, ANAPC5, DLGAP5, LIG1, PSRC1, CCNF, CENPF, CDC20, BIRC5, ESPL1, PBK, RACGAP1, TACC3, DCTN1, NCAPD2, CDC25B, FSD1, CHMP1A, CCNB2, PSMD12, PLK1, CALM3, AKAP8 |
| GO:0022402~cell cycle process | 41 | 2.13E-11 | PSMB10, XRCC2, PRC1, AURKA, CDC34, AURKB, GTSE1, SPC24, FAM83D, RPA1, CDCA8, SAC3D1, CDKN2D, BUB1, PSMD2, H2AFX, TCF3, CCNA2, NUDC, CUL1, CDCA3, MKI67, ANAPC5, DLGAP5, CCNF, CENPF, CDC20, BIRC5, ESPL1, PBK, RACGAP1, TACC3, DCTN1, NCAPD2, CDC25B, FSD1, CHMP1A, CCNB2, PSMD12, PLK1, AKAP8 |
| cell cycle | 31 | 1.16E-08 | E2F2, LZTS1, PRC1, AURKA, AURKB, CDT1, SPC24, FAM83D, CDCA8, CDKN2D, BUB1, H2AFX, CCNA2, NUDC, CDCA3, MKI67, ANAPC5, LIG1, DLGAP5, CCNF, CENPF, BIRC5, CDC20, RACGAP1, CDC25B, NCAPD2, FSD1, CHMP1A, CCNB2, PLK1, BLCAP |
| GO:0051301~cell division | 25 | 2.05E-08 | PRC1, AURKB, FAM83D, SPC24, CDCA8, SAC3D1, BUB1, CCNA2, NUDC, CDCA3, ANAPC5, LIG1, CCNF, CENPF, BIRC5, CDC20, ESPL1, RACGAP1, NCAPD2, CDC25B, FSD1, CHMP1A, CCNB2, PLK1, RAB35 |
| GO:0005819~spindle | 18 | 3.18E-08 | PRC1, DLGAP5, PSRC1, CENPF, AURKA, CDC20, BIRC5, AURKB, RACGAP1, DCTN1, FAM83D, CDCA8, SAC3D1, PLK1, BUB1, CALM3, MAP7D1, EMD |
| cell division | 22 | 6.93E-08 | PRC1, ANAPC5, LIG1, CCNF, CENPF, CDC20, BIRC5, AURKB, RACGAP1, CDC25B, NCAPD2, SPC24, FSD1, FAM83D, CHMP1A, CDCA8, CCNB2, PLK1, BUB1, CCNA2, NUDC, CDCA3 |
| mitosis | 18 | 1.37E-07 | ANAPC5, CCNF, CENPF, CDC20, BIRC5, AURKB, CDC25B, NCAPD2, SPC24, FSD1, FAM83D, CDCA8, CCNB2, PLK1, BUB1, CCNA2, NUDC, CDCA3 |
| GO:0015630~microtubule cytoskeleton | 34 | 2.41E-07 | DYNC1LI2, PRC1, KEAP1, AURKA, AURKB, GTSE1, TUBB4Q, FAM83D, CDCA8, SAC3D1, BUB1, SNTB2, TOP2A, EMD, NUDC, GABARAPL1, DLGAP5, PSRC1, CENPF, ESPL1, BIRC5, CDC20, RACGAP1, DCTN1, CDC25B, FSD1, EML3, CHMP1A, CCNB2, PLK1, CALM3, TUBA4A, MAP7D1, KIF20A |
